# Risk Intelligence: Making Profit from Uncertainty in Data Processing System

**DOI:** 10.1155/2014/398235

**Published:** 2014-04-24

**Authors:** Si Zheng, Xiangke Liao, Xiaodong Liu

**Affiliations:** School of Computer, National University of Defense Technology, China

## Abstract

In extreme scale data processing systems, fault tolerance is an essential and indispensable part. Proactive fault tolerance scheme (such as the speculative execution in MapReduce framework) is introduced to dramatically improve the response time of job executions when the failure becomes a norm rather than an exception. Efficient proactive fault tolerance schemes require precise knowledge on the task executions, which has been an open challenge for decades. To well address the issue, in this paper we design and implement RiskI, a profile-based prediction algorithm in conjunction with a riskaware task assignment algorithm, to accelerate task executions, taking the uncertainty nature of tasks into account. Our design demonstrates that the nature uncertainty brings not only great challenges, but also new opportunities. With a careful design, we can benefit from such uncertainties. We implement the idea in Hadoop 0.21.0 systems and the experimental results show that, compared with the traditional LATE algorithm, the response time can be improved by 46% with the same system throughput.

## 1. Introduction

Nowadays we are witnessing extreme scale data processing systems that employ thousands of servers to coordinately accomplish jobs [1]. In systems of such a large scale, hardware failure becomes a norm rather than an exception and the fault tolerance becomes an indispensable part of the system scheduling [2]. For example, in an early report in March 2006, Google reported that at least one server would fail every day, and each job would experience five failures. Besides, a single failure on job could cause completion time to increase by up to 50% [3, 4]. When the system scales, the problem becomes even more severe.

In traditional approaches, the fault tolerance is provided in a reactive manner. Failed jobs are reexecuted until they succeed. As the systems scale up, this simple reactive scheduling scheme becomes inefficient because the failures will become increasingly more frequent. In the extreme case, job may be executed forever due to continuous failures.

To well address this issue, a more promising approach is the proactive fault tolerance scheme. For example, in the MapReduce computational model [5, 6], the speculative execution is employed. Speculative execution will monitor the execution status of the subjobs (called tasks) and predict the execution failures. Once a task is obviously slower than the others (called straggler [6] or outlier [7]), a backup task will be executed to ensure the successful execution of the task. Similar proactive mechanisms are employed by other large scale data processing systems such as Mesos [8], Dryad [9], and Spark [10].

Speculative execution can dramatically reduce the execution time of tasks and bring great advantages on system response time. It is, however, on the cost of system throughput. To allow speculative executions, certain amount of the system resources will be allocated to execute backup tasks rather than new tasks, demoting the system throughput that depends on the allocated resources. In other words, there is a fundamental tradeoff between the overall system throughput and the response time of individual tasks. A well designed fault tolerance scheme should strike the best tradeoff between the two objectives.

Efficient fault tolerance schemes raise great challenges to the system designers. First, the optimal scheduling requires the precise knowledge on the execution time for each task [5, 6], which is very difficult, if not impossible in practice [11]. Robust scheduling algorithms can tolerate the inaccurate execution time information, but on the cost of the scheduling optimality. The widely adopted virtualization technology (e.g., [12]) makes the problem even more challenging.

Second, our later empirical experiments (in Section 3) will demonstrate that the task execution time in such a large scale is not a precise time but naturally contains uncertainties. It can only be confined to a range of time even when the same task is executed in the same environment.

Third, assuming the execution time is available for every task, a slower task is not necessary the straggler because of the different launch time of tasks [5]. The very nature of the MapReduce environment makes trouble in finding correct stragglers. The successful identification of stragglers requires much more design intelligence [6].

To well address these challenges and provide an efficient fault tolerance scheme, in this paper we propose a novel approach that exploits the very nature of the MapReduce-like large scale data processing systems. Our approach, called RiskI, is inspired by two observations. First, in such extreme scale data processing systems, jobs are periodically executed and thus we can collect the history information of a task to predict its future execution time. Second, the task execution time is naturally uncertain, and thus we can explore the risk management theory to assign tasks. For example, a faster node in average is not necessary to be a better node unless it can reduce the execution time for the whole job. To summarize, the main contributions of this paper are as follows.

First, we collect real traces from a MapReduce production system and find the uncertain nature of task execution time in such systems. We conduct comprehensive experiments to investigate the impact factors of the task uncertainties.

Second, we define a similarity measure to quantify the difference between executed tasks in system, based on which we design a task execution time prediction algorithm (Algorithm 1). Note that the output of the prediction is a range of time with probability distribution rather than a precise time.

Third, being aware of the task execution uncertainty, we design a risk management based task assignment algorithm and implement it on top of a Hadoop 0.21.0 production system. We conduct comprehensive experiments to evaluate the performance. Compared with the state-of-art scheduling algorithm LATE [6], the job response time can be improved by up to 46% with the same system throughput and compared with the native MapReduce scheduling with no fault tolerance capability, the performance degradation of our scheduling is neglected small.

The remainder of the paper is organized as follows. In Section 2, we give a review of the related works on performance prediction and task scheduling in distributed systems. In Section 3, we first introduce the background information of modern extreme scale data processing system and motivation of this work. In Section 4, we present our empirical study results revealing the very nature of the task execution uncertainty. We present our design on task execution time prediction and the risk management based scheduling in Sections 5 and 6, respectively. We will present the performance evaluation in Section 7 and draw a conclusion with the future work directions in the last section.

## 2. Related Work

Fault tolerance and task assignments have been widely studied, especially in traditional distributed system [13]. Research topics mainly include performance modeling and prediction [11], task assignment [14], heuristic algorithm [15], resource allocation [8], and fairness [16]. In our work, we mainly focus on two aspects, namely, the performance prediction and task assignment.

Performance modeling is a conventional way of predicting the execution time of tasks in advance of executions [17]. The design philosophy is to have a comprehensive understanding on machine capabilities and application features so that the execution behavior of the machines can be well characterized. Snavely et al. [11] proposed a framework for performance modeling and prediction on large HPC system. Their work focused on characterizing machine profiles and application signatures. Prediction is made by a proper convolution method. Modeling-based prediction works well under the condition of comprehensive understanding the machines and applications, complicated convolution method. These factors above may have the effect on prediction accuracy. Some works employed queuing network models [18–21] to represent and analyze resource sharing systems. The model is a collection of interacting services centers representing system resources and a set of customers representing the users sharing the resources. Another model is a simulation model [22] and it is actually the most flexible and general analysis technique. The main drawback is its development and execution cost. Petri nets can also be used to answer performance-related questions since they can verify the correctness of synchronization between various activities of concurrent systems [20, 23, 24].

Our work differs significantly from this literature as we make our prediction based on historical execution record. Because of considering a heterogeneous environment in MapReduce, getting such information is much costly and difficult. First, collecting and analyzing sufficient characterizations of the ability of the machine are an inefficient work. It needs to run low-level benchmarks for gathering performance attributes. It may be applicable for HPC system where the environment is homogeneous, but it is not practical in practical heterogeneous environments. Second, virtualization in computer makes some resource sharing invisible. Third, capturing and analyzing application features will introduce extra overhead. So, it is rarely possible to employ modeling-based method to predict execution time of task in MapReduce anymore.

Task assignment problem is also a very active research area, especially in traditional distributed system [25–28]. Much of works leverage precise time prediction for scheduling decision making [29, 30]. Other works concentrate on the target of load balance [31, 32]. However, in heterogeneous environment, the blind pursuit of load balance does not bring better performance any more.

Real-time scheduling [29] is relative work. Most works employ the worst time for scheduling to meet the requirement of real time and avoid the loss of uncertainty. Our work is also related to coscheduling [30] in multicore system, particularly with processor heterogeneity. Jiang et al. proposed a scheduling police based on prior knowledge that accurate threads execution times were known to the system. With this advanced knowledge, they can make better scheduling decisions. In contrast, our scheduling has no such precise and global information. Instead of making scheduling decisions based on precise time estimation, we take into account the fact that our prediction is a range of time.

Task assignment problem in MapReduce is still in the beginning since MapReduce adopted a simple scheduling police which is easy to implement. The master will assign a task to a tasktracker without considering its ability and possible execution time. It is only to lunch the task as early as possible. Run-time stragglers have been identified by past work. For the sake of reducing the response time of job, LATE [6] schedules a duplicate task for the straggler, which is named as speculative execution. However, since no distinction is made between tasktrackers, LATE cannot guarantee the speculative execution to be successful. So the effect of speculative execution is by chance totally. Delay scheduling [33] is another closed work we know of to our own. They begin to distinguish the tasktrackers with data locality. It schedules task as far as possible to the tasktracker who meets the need of data locally. Mantri [7] uses the progress rate instead of the task duration to remain agnostic to skews in work assignment among tasks.

Our work is different from above as we recognized the distinction of performance between working nodes. First, we investigated how to estimate the response time for a task on different working nodes. Instead of modeling-based method, we make our prediction through learning from experience. Second, since our prediction is a range of time which combines with confidence interval, we employed the method from risk of decision making to help us make scheduling decisions.

## 3. Motivation

In this section, we provide some background information of the MapReduce-like computing environments as the background information. We first describe the working mechanism of the MapReduce and elaborate how the speculative execution mechanism works. With simple examples, we point out the limitations in existing systems.

### 3.1. MapReduce Background

MapReduce is a typical representative of the modern parallel computing environment that provides extreme scale support. Hadoop is an open source implementation of MapReduce. Besides MapReduce and Hadoop, there are many other similar MapReduce-like computing frameworks such as the Mesos [8], Dryad [9], and Spark [10]. All these frameworks share a common idea.

**Figure 1:**
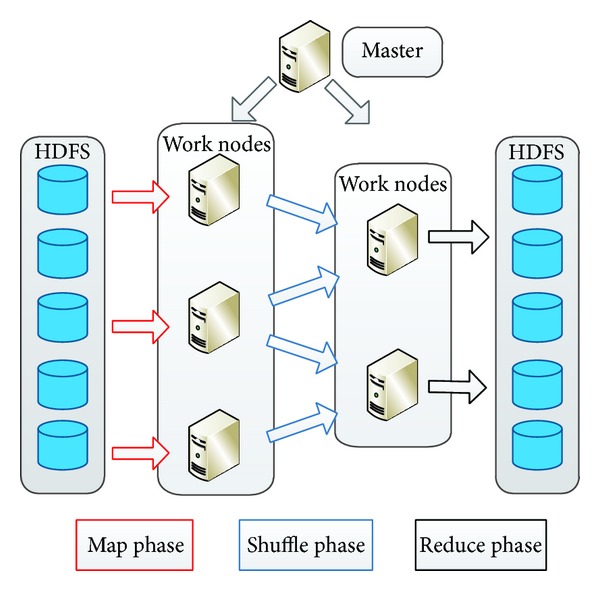
MapReduce computation framework that the job is split to two kinds of tasks, Map and Reduce. Different tasks are nearly independent so that different tasks can be executed in a parallel manner.

Roughly speaking, a computational system contains a master node and a number of working nodes. The master node splits the job to a number of nearly independent tasks and assigns them to working nodes. As these tasks are nearly independent, the parallelism can be fully exploited. As illustrated in Figure 1, a computation job mainly has three stages: the Map tasks for processing the raw data, the shuffle stage is to transfer the intermediate value, and the Reduce tasks that come out with the final results. Each working node has a number of slots for running the tasks. When a task finishes, the slot will be freed for the other task usage.

In extreme scale data processing systems, the traditional reactive fault tolerance scheme becomes ineffective because of the large number of failures and the long response. And thus, MapReduce framework adopts a proactive fault tolerance scheme. More specifically, when a task in execution is much slower than other tasks in the same job, speculative execution will be invoked to cope with this slowest task (called straggler [6]). In fact, because there is little dependence between tasks, the response time of a job is only determined by its straggler. To reduce the job response time and provide efficient proactive fault tolerance, we only need to accelerate its straggler while not delaying the other tasks too much so that new stragglers appear.

Speculative execution is based on a simple principle. When the straggler is identified, a backup task will be executed for the straggler, hoping the backup can be finished earlier than the straggler. As long as one wins, that is, either the original straggler or the backup one finishes, the other will be killed. It is essential to trade the system resources (and thus the overall throughput) to fuel the executions of individual jobs.

### 3.2. Challenges

In practice, efficient speculative execution faces many challenges. A crucial fact is that the backup task is not necessary to be the winner (finishes earlier). Speculative execution may fail, meaning that though the backup is invoked, the original straggler finishes earlier than the backup. When this happens, we in effect waste the resource allocated to backup task but make no benefit on response time. In other words, not all the speculative executions are profitable. We thus argue that the efficiency of the speculative execution highly depends on the wining rate of the backup tasks, defined as the percentage of the backup task that is finished earlier than the straggler. The main objective of this paper then becomes to increase the wining rate of backup tasks.

This is a very challenging issue in practice. First, stragglers can only be identified when they have been executed for a while, and thus the backup task starts always at later time. The backup must be sufficiently faster than the straggler to be the winner. This requires the accurate knowledge on the execution time of both stragglers and backup tasks. In literature, however, accurate prediction for task executions has been an open challenge for decades in traditional parallel systems. It is partially because, in traditional approaches [11], it is assumed that we have the comprehensive understanding of the machine capability and the job workload. This requires a great deal of measurement efforts, which are prohibitively high in a highly dynamic and heterogeneous environment as we are in. The application of virtualization makes the problem even challenging as resources become virtualized in an invisible manner. The real capability of each machine (virtual machine or virtual storage) is hard to measure.

Second, stragglers are difficult to identify. We are facing the dilemma that, on one hand, we desire an early identification of the stragglers so that the backup tasks have a larger improvement space and their wining becomes easier. On the other hand, later identifications of stragglers are more likely to be accurate as the stragglers may change during the execution.

Third, optimal speculative execution requires the precise execution time of tasks; for example, a task will be finished in 15 seconds. Unfortunately, uncertainty is a nature in MapReduce. The execution time of a task is within a range of time even under the same computing environments. Such an uncertainty is a double-blade sword. On the one hand, there will be no optimal assignment schemes in prior execution and thus to find the optimal one in advance is impossible. On the other hand, recall that the straggler determines the response time of jobs, and thus there will be multiple assignment schemes that perform exactly the same as the optimal assignment. In other words, even when our assignment scheme is not the optimal one, the performance may not be degraded. This property greatly eases the design challenges. In later sections, we will show how to benefit from this by applying the theory of decision making under risk and uncertainty.

## 4. Observation

In this section, we study the fundamental characteristics of the task execution in MapReduce environments. We first use experimental results to reveal the uncertainty nature of task executions, and then demonstrate the repeatable execution property of tasks. In the last, we will show that by a simple similarity-based scheme the degree of execution time uncertainty can be dramatically reduced.

### 4.1. Uncertainty of Task Executions

**Figure 2:**
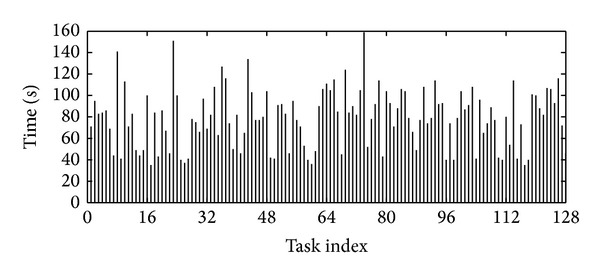
Execution time of tasks. There are 128 map tasks in total and each execution time of task was plotted.

In this part, we use experimental results to reveal the uncertainty nature of the MapReduce. We configure a standard Hadoop system version 0.21.0 (Hadoop is an open sourced implementation of MapReduce) with four machines and run a WordCount application [34]. In our experiments, 128 Map tasks and one reduce task are executed and the results are presented in Figure 2.

Figure 2 shows that the execution time ranges from 39 s to 159 s, with the average of 80 s. The standard deviation (Std) is 27.42 s. Note that the MapReduce system will equally split the Map tasks, and thus the workload of these 128 map tasks is nearly identical. We thus argue that uncertainty is a nature in MapReduce. On the other hand, even if identical tasks were executed in the identical machines or the same task repeatedly running under the same environment, the execution time is not identical but stable.  Figure 4(a) shows the CDF of response time of the task which was repeatedly running for 20 times in the same condition (we choose two tasks from two different jobs). We can figure that the execution time is also uncertain. By this nature, the traditional model-based task execution prediction algorithm is costly and will fail as the implicit assumption of this algorithm is that a task will behave the same under the same environment. However, we also find that the execution time is stable, which means most time is within a small range of time. So we argue that a reasonable prediction for the task should be a range of time with probability distribution.

### 4.2. Repeated Execution of MapReduce Tasks

In MapReduce environments, most of the jobs are batch for extreme scale data processing. The spirit is that moving the program is less costly than moving the data [5]. Consequently, the same jobs are repeatedly invoked to process different data [35]. For example, Facebook, Yahoo!, and eBay process terabytes of data and event logs per day on their MapReduce clusters for spam detection, business intelligence, and various optimizations. Moreover, such jobs rarely update. An interesting observation here is that the executions of a job in the past can be of great value for future. We can build profiles for each task and refer such profile to predict the job future executions.

Besides the similarity between tasks of different jobs, in some instance, similarity also exits in tasks of the same job. Because the number of tasks may be more than the number of slots, tasks may start and finish in “waves” [35].  Figure 6 shows the progress of the Map and Reduce tasks of a WordCount job with 8 GB data. The *x*-axis is the time and the *y*-axis is for the 34 map slots and 10 reduce slots. The block size of the file is 64 MB and there are 8 GB/64 MB = 128 input splits. As each split is processed by a different map task, the job consists of 64 map tasks. As seen in the figure, since the number of map tasks is greater than the number of provided map slots, the map stage proceeds in multiple rounds of slot assignment and the reduce stage proceeds in 3 waves. From the figure we can find that the tasks assigned to the same working node have the close execution time. The similarity exists between different waves on each node in map stage.

**Figure 3:**
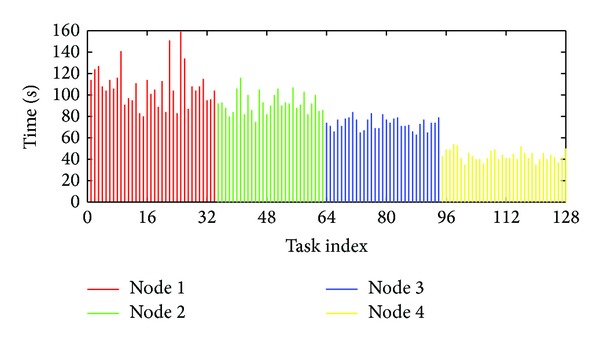
Reorganized execution time of tasks. Tasks in the same node were plotted together.

In Figure 2, we reorganize the execution time of tasks in Figure 3 while, this time, tasks executed in the same machine will be grouped together.  Table 1 gives the maximal and minimal average and Std of these tasks in each group. Compared with that in the original configuration, the Std reduces dramatically, for example, by 33% as from 27.42 to 18.46. This is because the four machines have the different hardware and tasks in different machines will experience the different resource sharing conditions. Nevertheless, tasks in the same machine will experience a more “similar” environment. This shows that a simple approach, with the similarity of tasks taken into account, can dramatically reduce the uncertainty. Next, we will show how to exploit such similarity.

It should be pointed out that the execution environment we mentioned above includes two parts. Except from the hardware configurations from different machines, tasks which are running parallel on the same execution unit (as known as tasktracker in Hadoop) can also affect execution environment. Based on different configuration, one or more tasks will run on the same tasktracker, which will cause resource contention and impact on execution time.

### 4.3. Design Overview

Keeping the uncertainty and tasks similarity nature in mind, in this paper we propose a novel proactive fault tolerance scheme in MapReduce environment. As illustrated in Figure 5, the design, named as RiskI, is constituted of two components: (i) a profile-based execution time prediction scheme and (ii) a risk-aware backup task assignment algorithm. The basic idea is that we build a profile for each executed task in history and refer the profile of a similar task (in terms of its own property and the environment) to predict a new task in execution. As the uncertainty is a nature that cannot be completely avoided, we design a risk-aware assignment algorithm that can benefit from such an uncertainty by exploring the theory of decision making under uncertainty. Our goal is to maximize the assignment profiting and minimize the risk meanwhile.

## 5. Execution Time Prediction

In this section, we will introduce how to leverage historical information to predict the execution time for tasks. We first present the architecture of the prediction algorithm and then introduce the components, respectively. In the last, we use a pseudocode to present details of the design.

### 5.1. Prediction Algorithm Architecture

The prediction of the job execution is based on a simple idea. For each executed task, we will maintain a profile to record its execution information. When predicting a new task, we refer to these historical profiles and look for the most similar task. Its execution time will then be our prediction result. Notice that due to the uncertainty nature of the job execution, the output of our prediction is also a range of time, indicating the maximum and minimum of the execution time, rather than a single value.

The prediction algorithm mainly consists of four steps, as illustrated in Figure 7. We first make a similarity search and look for the most similar task in history. Tasks will then be assigned and executed according to our predictions. As long as the execution is finished, we will adjust some critical control parameters and maintain the profiles for the next tasks. Noticing that there is no absolute the same between tasks, the key in the prediction algorithm design is the similarity of definition for any given two tasks that can yield the best prediction results. Next, we will give details on these steps.

### 5.2. Similarity Scheme

The similarity of two tasks can be affected by many factors such as the number of read bytes and the number of write bytes. In our work we apply all these factors, while our method has the adaptive capability that, for factors of little impact, they will be ignored automatically. This is done by applying an appropriate similarity scheme, that is, how to obtain the similarity based on these factors.

In literature, there are several approaches to measure the similarity that measures the similarity between tasks in different aspects. For example, the weighted Euclidean distance (WED) [36] measures the actual distance between two vectors, the cosine similarity measures the direction information, and the Jaccard similarity measures the duplication ratio. Considering the requirement of our work, we adopt WED and leave more design options for future work.

**Definition 1**

Given two tasks  *X* and  *Y*  with their affecting factor vectors  *x*_*j*_ and *y*_*j*_ and a weight scheme  *w*_*j*_, the similarity between the two tasks can be calculated as follows:

(1) $$\begin{array}{c} d\left(X,Y\right)=\sqrt[{2}]{\overset{n}{\underset{i=1}{\sum }}w_{i}{\left(x_{i}-y_{i}\right)}^{2}}, \end{array}$$

where  *n*  is the number of affecting factors and *w*_*j*_ is a weight scheme that reflects the importance of the different affecting factors on the similarity.

### 5.3. Weighting Scheme

Weighting scheme determines the impact of the affecting factors on the similarity measure. In practice, different machines may have different weighting schemes because of the different hardware. For example, for a CPU-rich machine, the IO may become the bottleneck of the task execution and therefore IO-related factors are more important, while IO-rich machines may desire CPU-related factors. To well address the issue, we apply a quadratic programming method to dynamically set the weighting.

Quadratic programming is a mature method for weight scheme setting in machine learning [15]. Our aim is to minimize the differences between the similarities calculated from our WED and the real differences obtained by comparing the real execution time. Supposing there are  *n*  task records in the node and each has  *q*  affecting factors, the constrains in the problem can be presented in formulas (2) and (3), in which  *S*_*ijk*_  is the similarity on the  *k*th attribute between task  *i*  and task  *j*, ∑_*k*=1_^*q*^ *S*_*ijk*_ *W*_*k*_  is the similarity between task  *i*  and task  *j*  calculated using formula (1), and *R*_*ij*_ is the real similarity between task  *i*  and task  *j*  calculated by real execution time. *L*_*ij*_ is the value by which the calculated similarity is less than the real similarity, and *M*_*ij*_ is the value by which the calculated similarity is greater than the real similarity:

(2) Minimize:  ∑i=1n∑j=i+1n(Lij2+Mij2)

(3) Subject  to:    ∑k=1qSijkWk+Lij−Mij=Rij (i,j=1…n,i<j)

(4)  Rij=1−min⁡{|t−tmax⁡|,|tmin⁡−t|}max⁡{t,tavg},

where *t* is the real execution time and the prediction time is  [*t*_min⁡_, *t*_max⁡_]. *t*_avg_ presents the average time of prediction.

As there is no comprehensive analysis of the complexity of quadratic programming problems, our analysis only can indicate how large this quadratic programming problem can be, but not the actual complexity of the problem. From the above formalization, the quadratic programming problem has  *n*∗(*n* − 1) + *q*  variables and  *n*∗(*n* − 1)  constraints.

The general factors used in calculating similarity include job name, node name, input size, workload, output size, and the parallel task that is running on the same node. We figure a rough weight scheme for job wordcount in one node. We showed it in Table 2. From the table we can see that the factor input size and IO-heavy task like randomwrite influence the execution time most.

## 6. Risk-Aware Assignment

In this section, we introduce our risk-aware assignment algorithm that attempts to benefit from the uncertainty of task executions. We first characterize the unique features and design overview of task assignment with uncertainty and then present the theory of decision making under uncertainty, which is the theoretical foundation for our risk-aware assignment. At last, we introduce our assignment algorithm based on the theory.

### 6.1. Design Overview

The method is presented as follows: in the first step, by comparing the average execution time of all the running tasks and the passed running time, we find out the straggler; in step two, we check out all the available work nodes and make the execution time prediction; in step three, we make the execution time prediction for the straggler running on the available node, in step four, with the help of the theory of decision making under risk, the most expected profiting assignment was chosen for speculative execution. If the new assignment can guarantee the speculative success, the original task can be killed.  Figure 8 is a flowchart of our risk-aware assignment design. The main challenge is how to leverage theory of risk decision to help us assign task.

### 6.2. Theory of Risk Decision

Since our prediction is a range of time, how to compare two ranges of time is another issue to help decision making. A direct method is comparing the average time of two ranges, and the distance of two average values is the time profit or time loss. The average time works well in the condition where time profit is uniformed per unit of time. However, this assumption is broken in MapReduce. As we mentioned in Section 3, because of the existence of straggler, the time profit is only related to the last finish time, but care nothing about the time before last finish time. In other words, may be some tasks would finish faster, it is still helpless for the execution time of the job, because the job would be finished after all the tasks were executed. Under this condition, time profit is different per unit time and the decision making under risk was introduced.

In many fields, risky choice and the selection criterion are what people seek to optimize. There are two parts included in risk of decision making, risk assessment and risk management. Risk assessment is in charge of evaluating risk or benefit and risk management takes responsibility for taking appropriate actions to control risk and maximize profit. Expected value criterion is also called Bayesian principle. It incorporates the probabilities of the states of nature, computes the expected value under each action, and then picks the action with the largest expected value. We can calculate the potential value of each option with the following equation:

(5) $$\begin{array}{c} E\left(X\right)=\underset{x}{\sum }P\left(x\right)U\left(x\right), \end{array}$$

where  *E*(*X*)  stands for the expected value. The probability  *P*(*x*)  of an event *x* is an indication of how likely that event is to happen. In the equation,  *U*(*x*)  denotes the profit and loss value of  *x*. The summation range includes every number of  *x*  that is a possible value of the random variable  *X*.

### 6.3. Profit Function and Unique Feature

First, the function  *P*(*x*)  is easy to get from historical statistic. As mentioned before,  *P*(*x*)  is the probability of the occurrence of state  *x*. In our case,  *P*(*t*_*i*_, *t*_*j*_)  represents the probability that the real execution time falls in the area between  [*t*_*i*_, *t*_*j*_]. This probability can learn from statistic information in history. For example, the output of prediction is a set  *T* = {*t*_min⁡_, *t*_*i*_,…, *t*_*j*_, *t*_max⁡_} in which the  *t*_*i*_  was sorted by its value. Since the prediction is a range of time with a probability distribution, we can exploit the probability distribution to compute the  *P*(*t*_*i*_, *t*_*j*_)  with the following equation:

(6) $$\begin{array}{c} P\left(t_{i},t_{j}\right)=\frac{\left|\left[t_{i},t_{j}\right]⋂T\right|}{\left|T\right|}, \end{array}$$

where  |*T*|  is the number of elements in the set  *T* and |[*t*_*i*_, *t*_*j*_] ⋂ *T*|  is the number of elements falling into range  [*t*_*i*_, *t*_*j*_].

Next, we will take an example to explain our definition for profit function  *U*(*x*). As shown in Figure 9(a), the execution time of task is a nature uncertainty and the assignment scheme should be aware of the potential risks. In Figure 9(b), because of the existence of straggler, the time profit is only related to the last finish time which was denoted as  *t*_*s*_. Under this condition, time profit is different per unit time and is divided into two parts which are part one  [*t*_*i*_, *t*_*s*_]  and part two  [*t*_*s*_, *t*_*j*_] and we plotted them in Figure 9(c). The profit function  *U*(*x*)  can be calculated as

(7) U(ti,tj)={avg(ti,ts)−ts[ti,ts]avg(ti,tj)−ts[ts,tj],

where avg(*t*_*i*_, *t*_*s*_)  is the average time of  [*t*_*i*_, *t*_*s*_] and avg(*t*_*i*_, *t*_*j*_) is the average time of [*t*_*i*_, *t*_*j*_].

Let  *T*  denote an assignment option. Let  *T*_1_  and  *T*_2_  denote two ranges of time divided by  *t*_*s*_. The expected profit can be calculated as

(8) $$\begin{array}{c} E\left(T\right)=P\left(T_{1}\right)U\left(T_{1}\right)+P\left(T_{2}\right)U\left(T_{2}\right). \end{array}$$

If  *E*(*T*) > 0,  *E*(*T*)  would be a better option since it is likely more close to reduce the completion time of a job. If there are more options for backup task assignment, the max  *E*(*T*)  would be the best choice.

A special case is that *t*_max⁡_ is less than the best time of straggler, as shown in Figure 9(d). In this case, the straggler can be killed immediately to release resources.

The detailed algorithm is shown in Algorithm 2. We first compare the average execution time of all the running tasks and the passed running time and then we find out the straggler. And then we check out all the available work nodes and make the execution time prediction. In step three, we make the execution time prediction for the straggler running on the available node. In step four, with the help of theory of decision making under risk, the most expected profiting assignment was chosen for speculative execution. If the new assignment can guarantee the speculative success, the original task can be killed.

## 7. Evaluation

We have conducted a series of experiments to evaluate the performance of RiskI and LATE in a variety combination of different job size. Since the environmental variability may result in high variance in the experiment result, we also performed evaluations on different heterogeneous environment configuration. We ran our first experiment on a small private cluster which has 5 working nodes and a master. Our private cluster occupies two whole Dell PowerEdge R710 machines. Each machine has 16 Xeon E5620 2.4 GHz CPUs, 12 GB DDR3 RAM, and  4 × 2.5*T*  disks. We use Xen virtualization software to manage virtual machines.  Table 3 lists the hardware configurations of our testbed. We installed our modified version of Hadoop 0.21.0 to cluster and the block size was configured as 64 MB. Different numbers of task slots were set to each node according to diversity of VM configurations.

### 7.1. Performance on Different Job Size

To identify the predominance of RiskI, we first ran some simple workloads. In this configuration, all jobs were set with one size in a round. We performed evaluation on three different types of job size, which are small, medium, and large, respectively. The small job only contains 2 maps, the medium job has 10 maps, and the large job has 20 maps. We chose Bailey-Borwein-Plouffe (BBP) job for evaluation and there are 20 jobs in total with arrival interval 60 s.

Figure 10(a) shows a CDF of job execution time for large jobs. We see that about 70% of the large jobs are significantly improved under our scheduling.  Figure 10(b) illustrates the corresponding running details, including the total number of speculative executions, those succeeded and being killed. From Figure 10(b), we can find that RiskI has two main benefits on scheduling. First, with the help of estimation, we are able to cut off some unnecessary speculative executions, thus release more resources. Since most speculative executions were failed in LATE, the total speculative executions were reduced from 181 to 34 in RiskI. Second, the successful ratio of speculative increased. In our scheduler only 6 speculative executions failed out of 34 and the success ratio is 81%.

Figures 10(c) and 10(d) plotted the results on medium job size and small job size. The performance on large job size is distinguished from those on smaller jobs because the small jobs cause less resource contention for low system workload.

### 7.2. Performance on Mix Job Size

In this section, we consider a workload with more complex job combination. We use Gridmix2 [37], a default benchmarks for Hadoop cluster, which can generate an arrival sequence of jobs with the input of a mix of synthetic jobs considering all typical possibility.

In order to simulate the real trace for synthetic workload, we generated job sets that follow the trace form Facebook that were used in the evaluation of delay scheduling [33]. Delay scheduling divided the jobs into 9 bins on different job size and assigned different proportion, respectively. For the sake of simplicity, we generalize these job sizes into 3 groups, small, medium, and large, respectively. Jobs in bin 1 take up about 38% in that trace and we assign the same proportion to the small jobs. We grouped bins 2–4 as medium jobs which also hold 38% in total. The rest of bins are grouped into large jobs with about 24% amount.  Table 4 summarizes the job size configuration.

Except for the job size proportion, arrival interval is also very important in generating a job arrival trace, since it may lead to different schedule behaviors and subsequently affect system throughput. In our experiment, we set the arrival interval to 60 s and 120 s.  Figure 11 gives the generated job sequences.

Finally, we ran the workload described above under two schedulers: LATE (Hadoop default scheduler FIFO) and RiskI. We submitted each job by only one user.

#### 7.2.1. Result for BBP

Our detailed configuration of BBP jobs was shown in Table 5.  Figure 12 plotted the execution time of each job with interval 60 s.

For ease of analysis, we divided the job sequence into 5 parts. The first part is from job 1 to job 13, where the jobs are medium. The second part is from job 14 to job 23. The jobs in the second part are all small. Job 24 to job 50 is the third part mainly consists of small and large jobs. The last 10 jobs are equally divided into two parts and the jobs are large and medium.

In the first part M, the response time of jobs is accumulated from the beginning, because the workload is becoming heavier gradually. The resource contention led to more stragglers and more speculative executions. More resources were used for backup task. The following jobs had to wait until resources were released. However, because of lack of delicate design of scheduling, most backup tasks failed. The system sacrificed the throughput but did not get benefit. From Figure 12(b) we can see that there are 48 backup tasks and only 19 succeeded. Compared to LATE, RiskI cut the number of backup task from 48 to 13. The static result was plotted in Figure 12(c). Many invalid speculative executions are avoided and saved the throughput. Since some backup tasks are certainly finished earlier, we killed the first attempt of task immediately in order to save resources. As a result, only 2 backup tasks failed. The same conditions occurred in the left 4 parts.

However, the situation is a little different when the job arrival interval is 120 s. We also plotted the execution time of each job with interval 120 s in Figure 13. There are little different exits in the first 4 parts. The reason is that the system workload is light because each job has 120 s before the next job arrives. The system has much free resources for backup task running. We can see from Figure 13(b) that no backup task succeeded in the first two parts. Even if the backup task failed, it will not cause any loss. But in RiskI, no backup task was launched for speculative execution which was plotted in Figure 13(c). Because none of them would succeed, most differences lied in the part L where we reduced the speculative execution count from 39 to 12 and only 1 speculative execution failed.

As a result, our scheduler performs much better in interval 60 than interval 120.

#### 7.2.2. Result for Streamsort

We evaluated the job of streamsort by running configured gridmix2 also. But we have to modify the Hadoop 0.21.0 for different arrival interval. The performance of sort is also improved better when workload is heavy since RiskI not only shortened the response time of job, but also killed some tasks to release resource earlier. When workload is light, our scheduler only takes advantage of reducing job response time.

Another conclusion is that the average performance of BBP is better than sort. The main reason is that, compared to BBP, prediction of task for streamsort has longer range which means more uncertainty.

### 7.3. Impact of Heterogeneous Degree

We change the testbed and Hadoop configurations to evaluate RiskI in different execution environments. We run our experiments in two other different environments. The original environment above is denoted as testbed 1. And the new environments were denoted as testbeds 2 and 3. The testbed 3 is 4 times more heterogeneous than testbed 2 and 8 times than testbed 1. We measure heterogeneity by its number of virtual machines on the same physical machine. The result was shown in Figure 14. When the environment is more heterogeneous, RiskI perform degrades much less than LATE.

### 7.4. Stable of Prediction

Since we make our prediction by searching similar executions of task in history, we have to demonstrate that the similar executions have stable response time. It is obvious that stable response time will make our prediction significant. Otherwise, our prediction is helpless for scheduling. Therefore, we did the following experiments to proof our prediction.

**Figure 4:**
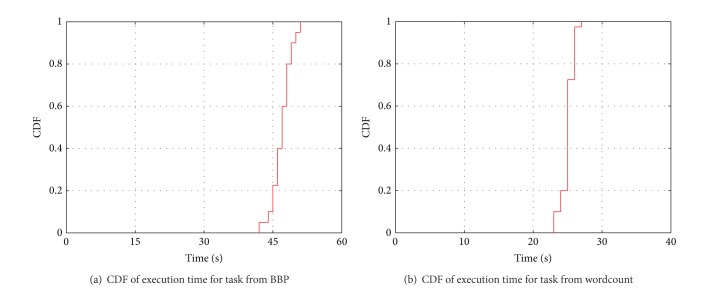
Execution time.

**Figure 5:**
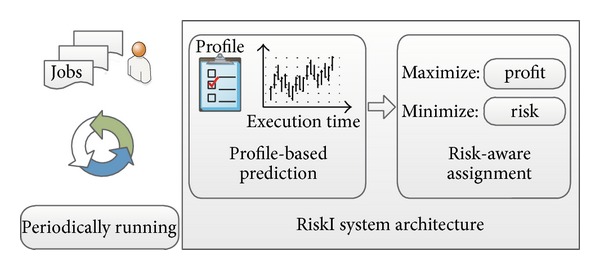
RiskI has two main parts, a profile-based execution algorithm for task execution time prediction and a risk-aware backup task assignment algorithm used to make scheduling based on range of time.

**Figure 6:**
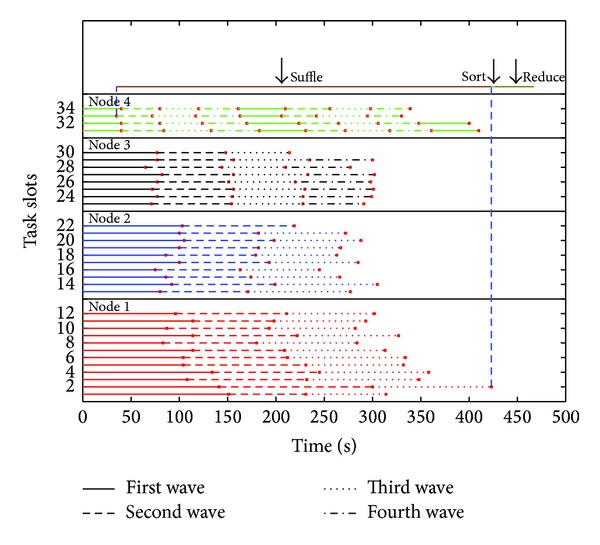
“Waves” in execution. Since the number of tasks is more than task slots, tasks were executed in “waves.” In our example, there are 9 waves in node 4 and 3 waves in node 1.

**Figure 7:**
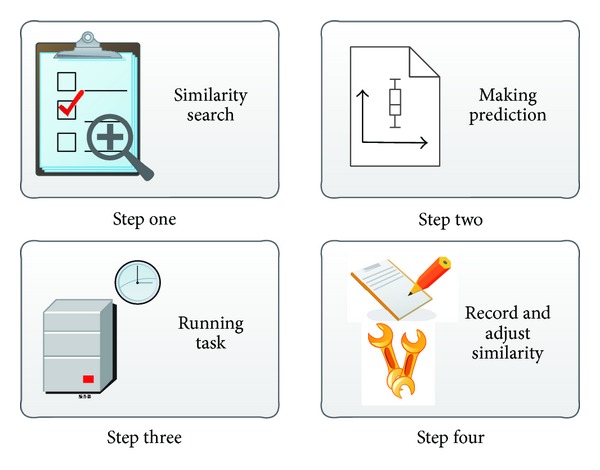
Our prediction has four steps. Upon a new task is in prediction, we firstly look for the most similar task in history and then make a prediction based on similar task. Then, we wait for the task running and record the running information and adjust weight setting scheme.

**Figure 8:**
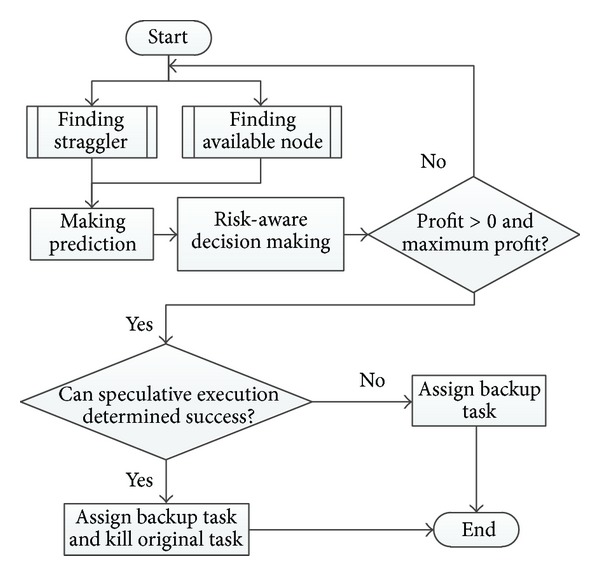
Flowchart of risk-aware assignment.

**Figure 9:**
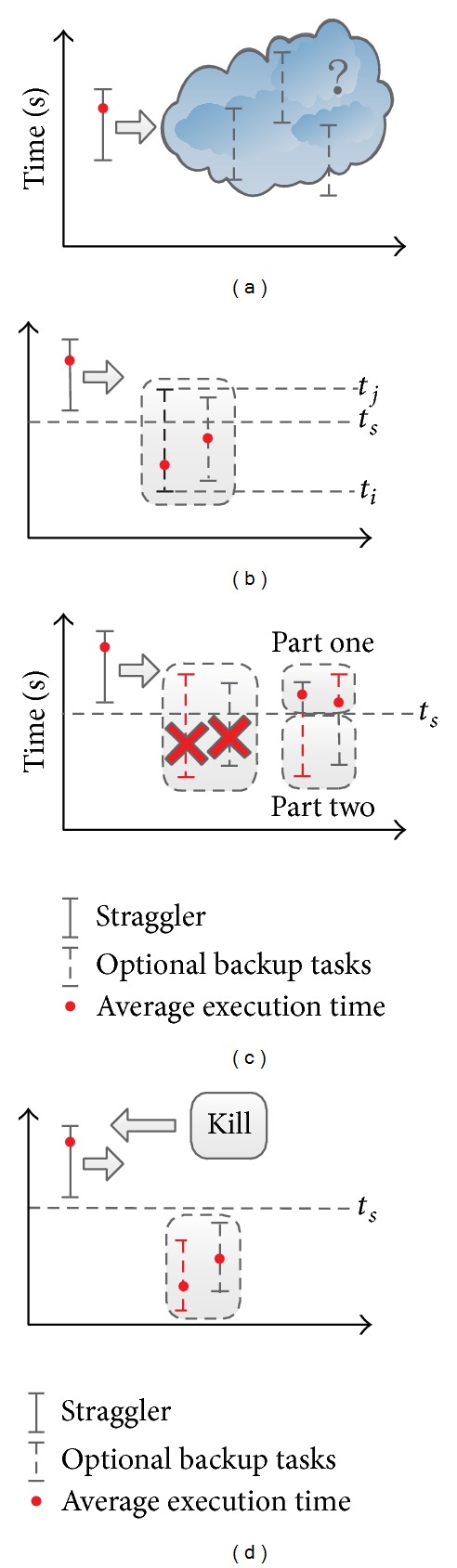
An example for different time profit per unit. The time profit is divided into two parts. So we considered separately these two parts.

**Figure 10:**
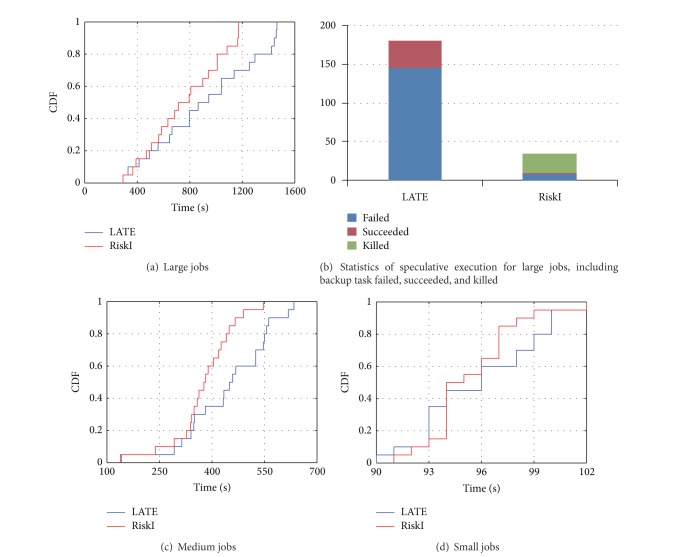
CDFs of execution times of jobs in various job sizes. RiskI greatly improves performance for all size of jobs, especially for large and medium jobs.

**Figure 11:**
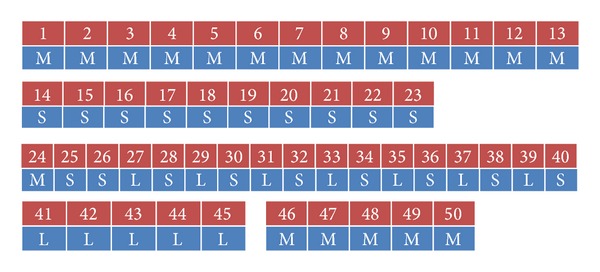
The Gridmix job has 50 jobs in total. The first 13 jobs are medium size. The jobs from 14 to 23 are small. The small job and large job are alternate arrived from job 26 to job 40. The large jobs appear consecutive form job 41 to job 45 and the last 5 jobs are medium.

**Figure 12:**
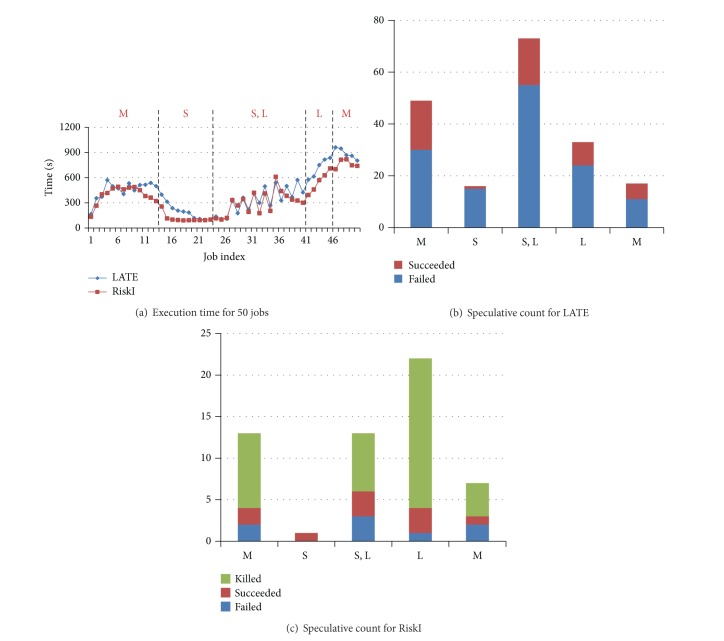
Details of execution information with interval 60. (a) is static of execution time for each job. (b and c) are statistics of speculative execution count in different parts.

**Figure 13:**
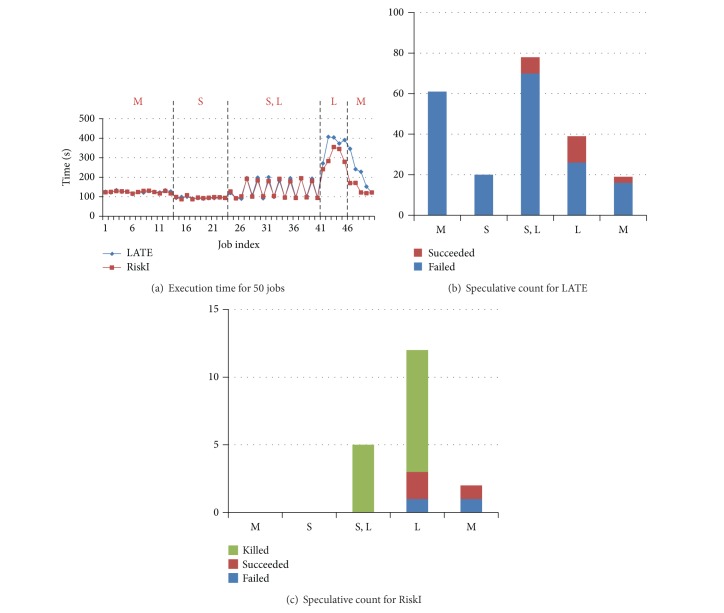
Details of execution information with interval 120. (a) is static of execution time for each job. (b and c) are statistics of speculative execution count in different parts.

**Figure 14:**
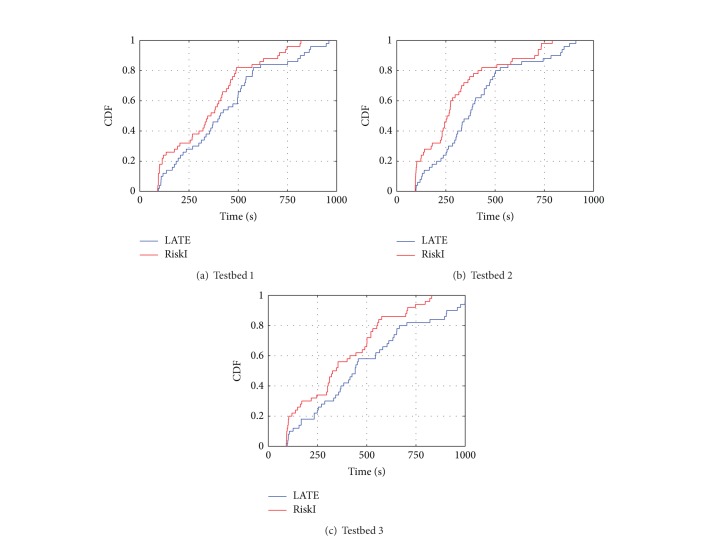
CDFs of execution time in different testbeds.

**Figure 15:**
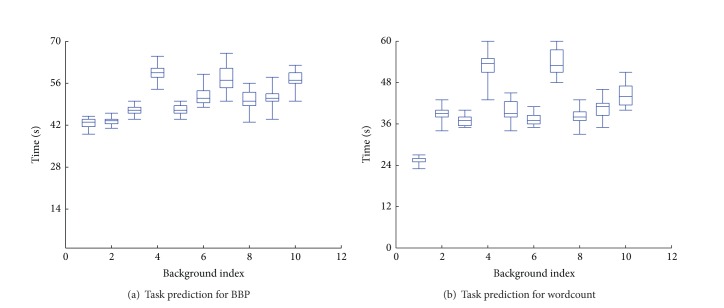
Execution time prediction.

First, we selected a four-slot work node which implies at most four tasks that can run on the node in parallel. And then we investigated the task running time under all the possible execution conditions. These conditions consist of various combinations of tasks which are selected from wordcount, randomwrite, and Bailey-Borwein-Plouffe. So there are 9 conditions in total.  Figure 15(a) shows our results on running time of task from BBP job that the task ran 20 times in every environment. We can see that the execution time is relatively stable in a range time. For further analysis, Figure 4(a) shows the CDF of response time distribution between the longest time and the shortest time in one condition. It demonstrated that most times are centralized distribution. The result of task from wordcount is shown in Figures 15(b) and 4(b). The conclusion can be also certified.

**Figure 16:**
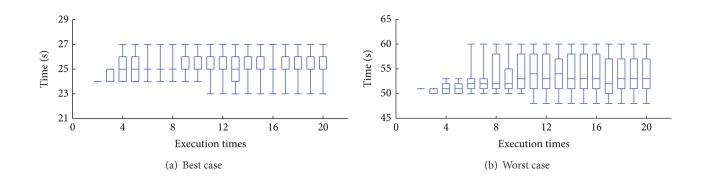
Prediction evolution.

We showed the prediction evolution with the execution times increased in Figure 16. We selected two conditions where the prediction is most stable and most unstable. The evolutionary process of prediction is plotted.

## 8. Conclusion

We propose a framework named RiskI for task assignment in a fault tolerance data processing system. We suggest that, by finding the most similar task in history, profile-based method can predict the execution time with a range of time. Then it makes risk-aware assignment for task to seek more profit while reducing the risk introduced by uncertainty. Extensive evaluations have shown that RiskI can provide good performance in all conditions. And RiskI performs much better when the system puts up more heterogeneity. We believe that the performance of RiskI will improve further if we understand the behavior of system and find better similar algorithm.

## Figures and Tables

**Algorithm 1:**
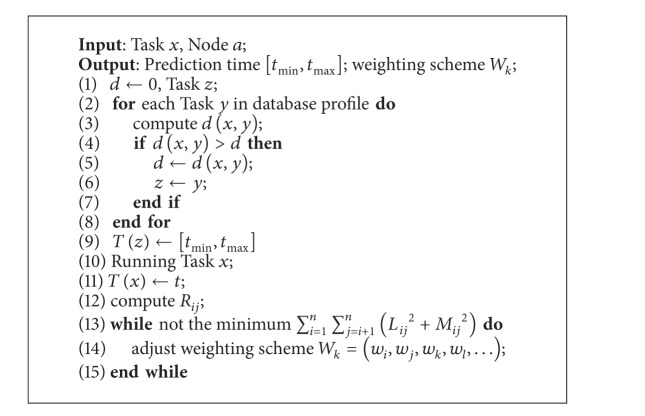
Algorithm for task execution time prediction.

**Algorithm 2:**
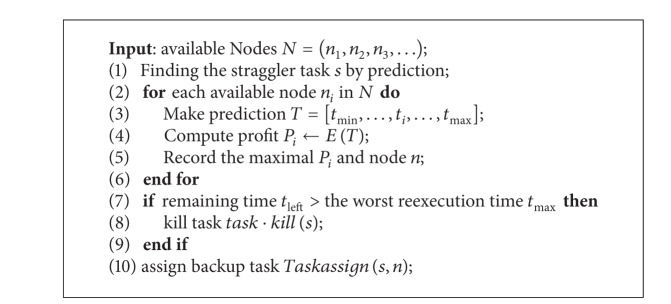
Algorithm for risk-aware assignment.

**Table 1:** Execution time statistics for 128 map tasks from job wordcount.

	Avg.	Std	Max.	Min.
Total	80	27.42	159	32
Node 1	108.61	18.46	159	80
Node 2	92.66	9.68	116	75
Node 3	73.61	5.61	84	63
Node 4	43.5	4.94	54	32

**Table 2:** A rough weight scheme for job wordcount.

Factors	Weight
Job name	
Work node	
Input size	34.5%
Environment	
Task 1 (randomwrite)	33.6%
Task 2 (wordcount map)	15.8%
Task 3 (BBP)	14.1%
Others	2%

**Table 3:** Our testbed used in evaluation.

VMs	Map slots	Reduce slots	VM configuration
Node 1	0	1	1 core, 2 G RAM
Node 2	4	0	4 core, 2 G RAM
Node 3	4	4	4 core, 2 G RAM
Node 4	4	4	4 core, 2 G RAM
Node 5	1	1	4 core, 2 G RAM

Total	13	10	17 core, 10 G RAM

**Table 4:** Distribution of job sizes.

Job size	# Map	# Reduce	# Jobs	% Jobs
Small	2	2	19	38%
Medium	10	5	19	38%
Large	20	10	12	24%

Total	468	253	50	

**Table 5:** The configuration details for BBP, including start digit, end digit, and the workload per map task.

Job size	Start Digit	ndigit	Workload/map
Small	1	25000	156283174
Medium	1	55000	151332558
Large	1	70000	122287914
